# Moderate effect of early-life experience on dentate gyrus function

**DOI:** 10.1186/s13041-022-00980-1

**Published:** 2022-11-21

**Authors:** Pacifique Rukundo, Ting Feng, Vincent Pham, Simon Pieraut

**Affiliations:** Department of Biology, University of Nevada, Reno, NV 89557 USA

**Keywords:** Dentate gyrus, Hippocampus, Plasticity, Memory discrimination, Postnatal development, Enriched environment, Pattern separation, Behavior

## Abstract

The development, maturation, and plasticity of neural circuits are strongly influenced by experience and the interaction of an individual with their environment can have a long-lasting effect on cognitive function. Using an enriched environment (EE) paradigm, we have recently demonstrated that enhancing social, physical, and sensory activity during the pre-weaning time in mice led to an increase of inhibitory and excitatory synapses in the dentate gyrus (DG) of the hippocampus. The structural plasticity induced by experience may affect information processing in the circuit. The DG performs pattern separation, a computation that enables the encoding of very similar and overlapping inputs into dissimilar outputs. In the presented study, we have tested the hypothesis that an EE in juvenile mice will affect DG’s functions that are relevant for pattern separation: the decorrelation of the inputs from the entorhinal cortex (EC) and the recruitment of the principal excitatory granule cell (GC) during behavior. First, using a novel slice electrophysiology protocol, we found that the transformation of the incoming signal from the EC afferents by individual GC is moderately affected by EE. We further show that EE does not affect behaviorally induced recruitment of principal excitatory GC. Lastly, using the novel object recognition task, a hippocampus-dependent memory test, we show that the ontogeny of this discrimination task was similar among the EE mice and the controls. Taken together, our work demonstrates that pre-weaning enrichment moderately affects DG function.

## Introduction

During postnatal development, experience-dependent mechanisms refine hippocampal circuits and affect both excitatory and inhibitory synapse formation [1–5]. This structural plasticity is thought to underlie behavioral changes and have a long lasting effect on cognitive functions [6–10]. The experience-dependent refinement of synapse number is considered, along with the changes in synaptic strength, to be the substrate for learning and memory, and enabling adaptation to new environments [11–13]. It is not clear, however, whether and to what extent these synaptic alterations support the tuning of the computational processes performed within a circuit. Using the enrichment housing paradigm in which juvenile mice are raised in large cages with toys, a running wheel, and diverse objects, we have recently shown that the experience-dependent plasticity of the hippocampal synaptic network is not homogeneous but rather affects specific microcircuits [3]. In the dentate gyrus (DG) of mice raised in an enriched environment (EE), we found a robust increase in the inhibitory drive onto principal excitatory cells which was primarily mediated by the addition of somatic GABAergic synapses from the cholecystokinin expressing (CCK +) interneurons (IN) [3]. The specificity of this structural plasticity is quite remarkable, underscoring a key role for this cell type in adjusting inhibition during development and possibly the computation in DG circuit [3]. The DG circuit is the gateway to the hippocampus, which plays an essential role in spatial navigation, social cognition and, learning and memory. The circuit processes inputs from the entorhinal cortex (EC) and enables encoding of very similar inputs into dissimilar outputs, a computational process known as pattern separation [14–16]. Pattern separation minimizes interferences between inputs and is critical for sensory and memory discrimination [14, 17–19]. Pattern separation declines with aging and is compromised in psychiatric and neurological conditions such as Alzheimer disease, post-traumatic stress disorder and schizophrenia [20–26]. The ontogeny of pattern separation, which is the process to acquire this function during postnatal brain development, is not known. Considering that, for the most part, GC connectivity is hardwired [2], the emergence of this computation may be controlled by genetically encoded developmental programs. In light of our recent findings demonstrating experience-dependent remodeling of the inhibitory and excitatory networks in the circuit [3], it remains to be tested whether DG computation can be tuned in response to these plasticity mechanisms. In the present study, we asked whether DG computation is stable during postnatal development or adjusted in response to a change in the rodent’s environment. To address this question, we used a pre-weaning enrichment protocol in which mice are raised from birth to postnatal day 21 (P21) in either a standard housing cage (SH) or an EE cage and subsequently tested to assess DG computation. It is proposed that pattern separation takes place at two different levels: the neuronal population level and single neuron level. At the population level, pattern separation will affect the recruitment of distinct GC ensembles activated by similar, yet different stimuli; this is thought to permit the encoding of similar representations by distinct GC ensembles [16, 27]. At the single neuron level, individual GC transform the input pattern it receives into a dissimilar output pattern by changing firing rate and by orthogonalizing the spiking pattern [28–32]. Using a combination of electrophysiology and behavioral approaches, we tested whether our EE protocol affected DG computation at the single neuron and at the neuron population level. We found that *in-vitro*, the transformation of the incoming EC signal is moderately affected by EE. At the population level, we found that EE does not affect the recruitment of the GC during a novel object exploration task. Lastly, using the novel object recognition taks, we show that the ontogeny of this memory discrimination is unaffected by the EE. Taken together, these findings demonstrate that while the computation of the hippocampal circuit can be moderately tuned by experience-dependent mechanisms, the ontogeny of the DG function is preserved at juvenile age.

## Results

**Fig. 1:**
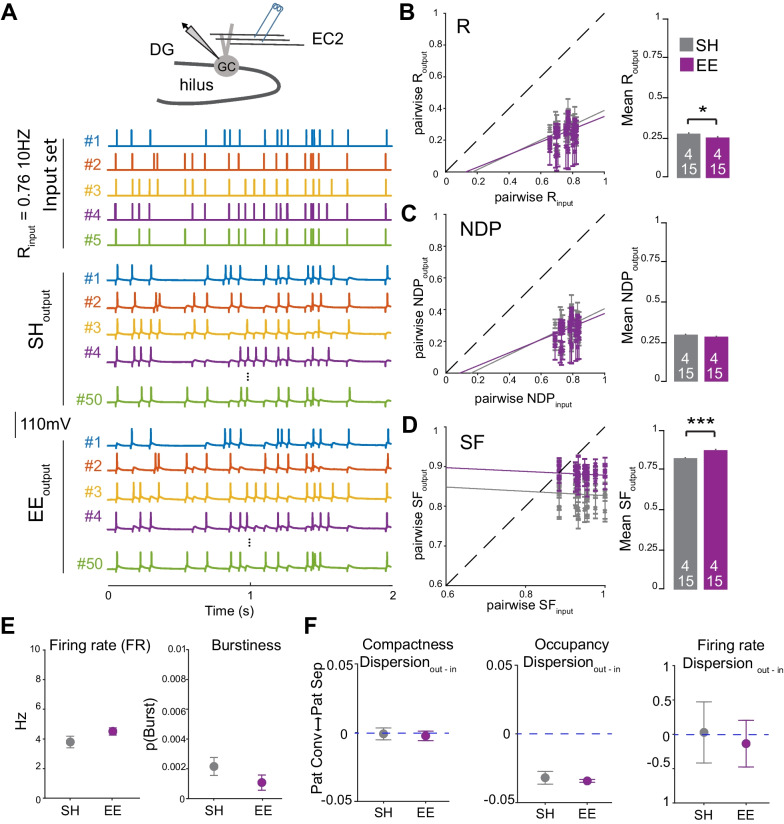
Pre-weaning enrichment affects spiketrain transformation in the DG. **A** Top, cartoon depicting whole-cell recording of the GCs during simultaneous stimulation of the perforant path (EC afferents). Bottom, representative traces obtained in current-clamp mode in response to the stimulation with 5 input spike trains. To assess the level of pattern separation operated by single recorded GCs, the similarity of the inputs is compared to the similarity of the outputs using different comparative metrics. **B** − **D** Representative graphs of pattern separation with pairwise output similarity versus the pairwise input similarity measured by the three different metrics, including R (**B**), NDP (**C**) and SF (**D**) and using 10 ms bins. Data points below and above the dashed line correspond to pattern separation and pattern convergence, respectively. Solid lines represent the linear fits for the ANCOVA. Statistical comparisons were performed with an ANCOVA (the aoctool function in Matlab) and a two-sample t-test (*p < 0.05 and ***p < 0.001). **E** A summary for FR and p(Burst). Statistical comparisons were performed with a two-samples t-test for firing rate (t = 1.559, p = 0.13) and p(Burst) (t = 1.3627, p = 0.184). **F** Compactness, Occupancy or FR codes were measured for each recording set. Statistical comparisons were performed with 2 sample t-test for binwise Compactness of output (t = 1.04, p = 0.307), the variations of Occupancy (t = 1.924, p = 0.073) and the variations in FR or spike trains (t = 1.129, p = 0.269). Number of animals = 4 for both groups and a total of 15 recordings were used for each group for the spiketrain analysis

To test our hypothesis that an EE alters pattern separation in the DG, we use an in-vitro slice electrophysiological approach recently established by Madar and colleagues [31]. This protocol compares the similarity of the incoming signal (input spike trains) delivered to the DG with the similarity of the recorded output spike trains. We performed whole cell recording on single GCs located in the upper part of the GC layer to monitor the spiking activity of the mature GC. Using a theta glass pipet, we stimulated EC afferent inputs with 5 distinct 10 Hz input spike trains and, simultaneously, recorded the output spike trains from the GC. The similarity of the input spike trains can then be compared with the similarity of the output trains (recorded from the GC) using multiple metrics, each of which inform about computational transformation performed by the DG (Fig. 1A) [31]. The spiking activity of the cells was first computed to calculate the output correlation (R_output_) using pairwise Pearson’s correlation coefficient (R). We found that in both SH and EE mice, R_output_ was lower that the R_input_, supporting a decorrelation of the incoming signal by DG. This is in accordance with pattern separation operated in this circuit. When comparing the mean R_output_ obtained from SH and EE mice, we found a statistically significant, yet small, decrease between the two groups (mean SH-R_output_ = 0.27, mean EE-R_output mean_ = 0.25, p = 0.029). Moreover, even though correlation coefficients of the R_output_ with the R_input_ are statistically different, they are very close between the two groups (Fig. 1B). Because pattern separation can be considered as having the inputs’ pattern being orthogonalized, we also computed the normalized dot product (NDP, cosine of the angle between two vectors) of both input and output patterns. This allowed us to assess the extent to which the signal, viewed as vectors of spike-counts, is transformed by the DG (i.e., “orthogonalized”). We found no statistical differences between SH and EE mice using the NDP metric (mean SH-NDP_output_ = 0.30, mean EE-NDP_output_ = 0.28, p = 0.14) (Fig. 1C). We then sought to compare the scaling factor value (SF) of the input and output spike trains, which enabled us to assess if the number of spikes per “bin” is scaled up or down. Our analysis demonstrated a statistically significant change of the SF_output_ values in the EE group compared to the SH group (mean SH-SF_output_ = 0.83, mean EE-SF_output_ = 0.88, p = 0.000) (Fig. 1D). Since SF, but not R or NDP, is correlated to the firing rate [32], we wanted to assess if the firing property of the outputs were different among the two groups. We first compared the firing rate (FR) and burstiness (pBurst) of the recorded neurons. Both pBurst, i.e. the probability of firing a small burst of AP following a single stimulus, and firing rate were identical in SH and EE groups (Fig. 1E). To further explore a putative change in spike train features, we calculated dispersion of the spike train firing rate, compactness, and occupancy (see material and method section for more details). We found no difference in these metrics suggesting that in response to the same input pattern, the output spike trains are highly similar in terms of pattern of spike distribution and firing structures (Fig. 1F). Together these data demonstrate that the pattern separation of spiketrain is moderately affected by housing conditions. Additionally, the change between the two groups for SF and R metrics argues that the computation can be tuned by experience-dependent mechanisms. EE induces an increased excitatory and inhibitory drive in the DG circuit [1, 3]. The increased excitatory drive can in turn reduce the sparsification of the signal in the DG by increasing the activity of the GC in response to EC imputs. An expected consequence would be an overall increase in the number of GC active during the behavioral task. On the other hand, the previously observed increase in CCK-IN inhibition induced by EE [3] is expected to maintain the excitatory to inhibitory balance and the overall sparsification of the EC inputs (e.g. sparse activity of the GC).

**Fig. 2:**
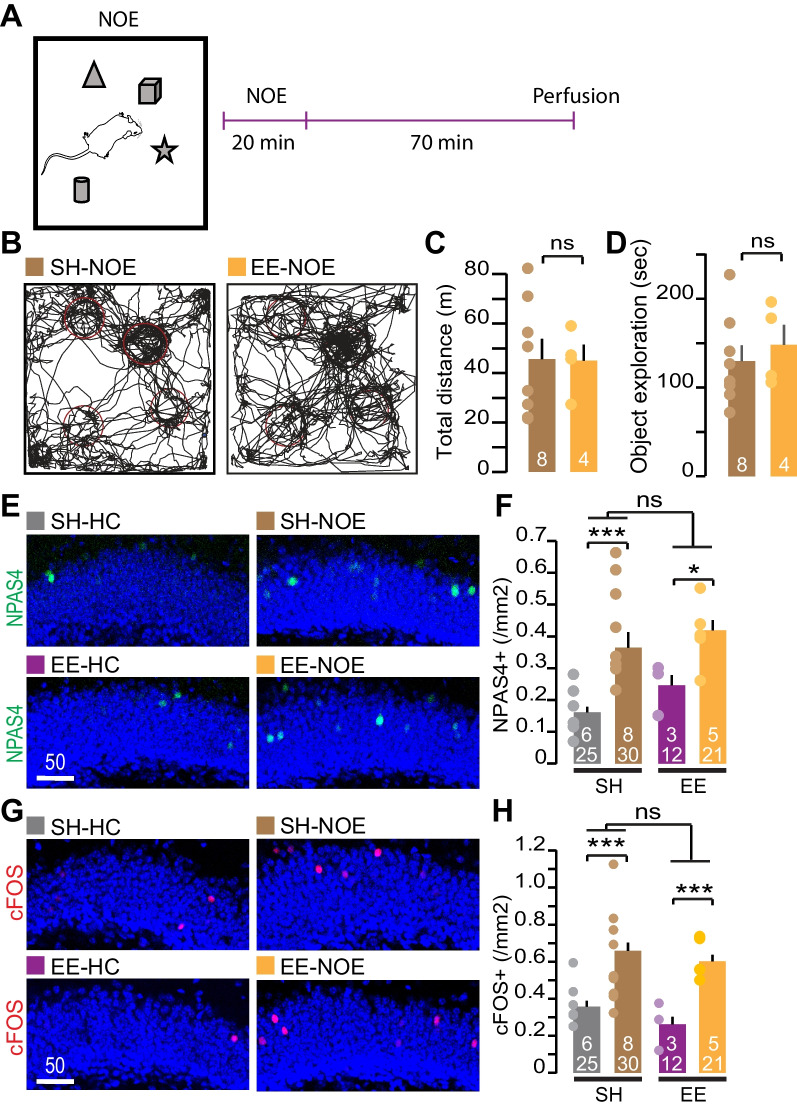
Pre-weaning enrichment does not affect GC recruitment during object exploration. **A** Schematic of the novel object exploration (NOE) experiment. **B** Representative video tracking traces of an individual SH and EE mouse performing the NOE (SH-NOE and EE-NOE respectively). **C** Average of the total distance in meters and **D** total time exploring the objects is reported. Two-sample t-test shows non-significant (ns) differences. **E**, **G** Representative images of NPAS4 and cFOS expression in the DG of SH-NOE, EE-NOE and control SH and EE mice kept in their home cages (HC) during the time of the experiment (e.g., mice that did not perform the NOE task: SH-HC and EE-HC respectively). **F**, **H** Expression of cFOS and NPAS4 was assessed 70 min following the NOE task and quantifications show an increased cFOS + and NPAS4 + GCs in SH-NOE and EE-NOE groups compared to their HC littermate (SH-HC and EE-HC). Non-significant (ns) differences in the densities of cFOS + and NPAS4 + were found in the EE groups compared to SH groups as shown by two-way ANOVA analysis (NPAS4 p = 0.366, cFOS p = 0.181). Asterisks represent 2-way ANOVA post-hoc comparison *p < 0.05, ***p < 0.001. Scale bar in µm. For each bar, the number of animals (on top) and the number of images (on the bottom) used for the quantification are reported. Individual dots represent the mean per animal

To address whether the recruitment of GC *in-vivo* is sensitive to early-life experience, we tested how EE preconditioning affected the number of active GCs during exploratory behavior. SH and EE mice were placed in a novel environment containing 4 objects for 20 min (Fig. 2A). After this novel object exploration task (NOE), the mice were euthanized for immunohistochemistry (IHC). The recruitment of GC during NOE was assessed by calculating the number of GCs positive for cFOS and NPAS4 protein expression. cFos and NPAS4 are immediate early genes (IEG) downstream of neural activity, thus their expression reports neural activation [33–38]. To prevent confounding factors due to change in exploratory behavior (the EE mice may be more gregarious and explore more than the control SH mice [10, 39]), we first assessed their behavior using a video tracking method. The pre-weaning enrichment did not affect novel object exploration per se since both groups ran the same distance and spent a similar amount of time exploring the objects during the task (total distance, mean SH-NOE = 45.56 m, mean EE-NOE = 44.88 m, p = 0.96; time exploring object, mean SH-NOE = 129.8 s, mean EE-NOE = 148.0 s, p = 0.55) (Fig. 2B–D). The total mobile time in the arena during the NOE task was also similar between the two groups (data not shown). We then quantified the density of cFOS + and NPAS4 + GCs in the GCL of brain sections harvested 70 min after the task. Previous studies have shown that the recruitment of GC during spatial exploration and learning tasks is very sparse [16, 18, 28, 40]. In accordance, we found a very low number of GCs expressing cFOS + and NPAS4 + in all conditions (cFOS + : mean SH-HC = 0.36 cells/mm^2^, mean SH-NOE = 0.66 cells/mm^2^, mean EE-HC = 0.26 cells/mm^2^, mean EE-NOE = 0.60 cells/mm^2^ and NPAS4 + : mean SH-HC = 0.16 cells/mm^2^, mean SH-NOE = 0.43 cells/mm^2^, mean EE-HC = 0.24 cells/mm^2^, mean EE-NOE = 0.42 cells/mm^2^). Two-way ANOVA analyses shows that the EE has no effect on cFOS and NPAS4 expression (p = 0.181 and p = 0.366 respectively). This result demonstrates that EE does not impact the expression of cFOS and NPAS4 induced by NOE suggesting that the recruitment of the GC during this exploratory behavior is identical in both groups.

**Fig. 3:**
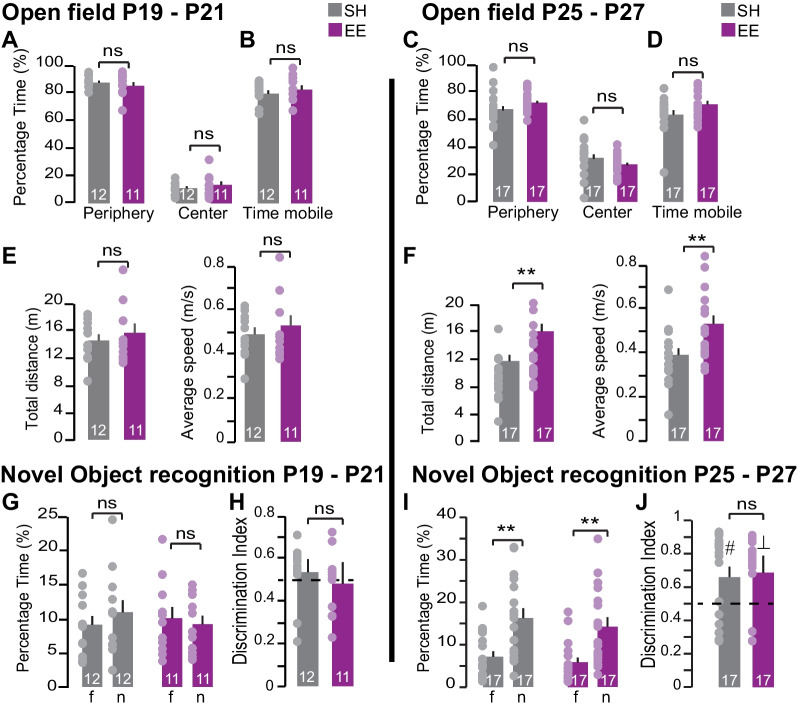
Pre-weaning enrichment does not affect the ontogeny of novel object recognition. **A**–**F** Open field (OF) locomotor activity analysis and **G**–**J** novel object recognition (NOR) memory test was performed using video tracking in SH and EE mice grouped by age: **A**, **B**, **E**, **G**, **H** P19–P21 and **C**, **D**, **F**, **I**, **J** P25–P27. **A**, **C** Total distance in the peripheral sector and the center of the arena during OF (**B**, **D**) Total time the mice are mobile during the OF test for each age group. **E**, **F** Total distance and average speed of the SH and EE mice during the OF test for each age group. **G**, **I** Time spent exploring both familiar (f) and novel (n) objects for each age group during the NOR test. In both SH and EE mice age P25–P27, mice spent more time exploring novel object compared to the familiar. **H**, **J** Discrimination index (DI) calculated as time spent exploring novel object divided by total object exploration time. DI values are non-significantly different between SH and EE mice in both age groups. A preference for novel object is seen in older juvenile (P25–P27) as demonstrated by one-sample t-test above chance (0.5) (#) p = 0.01 and ($\bot$) p = 0.0027. All other statistics were done using a two-sample t-test (**p < 0.01). For each bar, the number of animals used for the quantification is reported; individual dots represent the mean per animal

The behavioral analysis of the mice performing NOE shows that EE has limited effect on the animal’s exploratory behavior (Fig. 2B, D). We performed additional analyses to assess potential behavioral difference between the SH and EE mice. An open field (OF) test was first used to assess novelty-induced psychomotor activity and anxiety-related behavior. We performed the test with groups of mice of different ages: one group with mice at age P19 to P21 and the other at age P25 to P27. We found consistent for both age groups, EE had no effect on the time the mice spent exploring either the center or the periphery of the arena (mean % time in periphery P19–P21: SH = 88.99%, EE = 86.61%, p = 0.41; mean % time in center P19–P21: SH = 11.01%, EE = 13.39, p = 0.041) (Fig. 3A) (mean % time in periphery P25–P27: SH = 68.03%, EE = 72.75%, p = 0.20; mean % time in center P25–P27: SH = 31.97%, EE = 27.25, p = 0.20) (Fig. 3B). The time the mice spent being mobile during the OF test was also similar in SH and EE mice (mean % time mobile P19–P21: SH = 80.84%, EE = 83.77%, p = 0.44; mean % time mobile at P25–P27: SH = 64.22%, EE = 71.85%, p = 0.081) (Fig. 3A, B). This suggests that novelty-induced anxiety is not affected by the EE pre-conditioning. At P19-21, the speed of the SH and EE mice and the distance they ran was similar (mean distance P19–P21: SH = 14.35 m, EE = 15.47 m, p = 0.48, mean speed: SH = 0.05 m/min, EE = 0.05 m/min, p = 0.47) (Fig. 3C) but at P25-27 these parameters were increased in the EE group compared to the SH mice (mean distance P25–P27: SH = 11.61 m, EE = 15.87 m, p = 0.005, mean speed: SH = 0.04 m/min, EE = 0.05 m/min, p = 0.006) (Fig. 3D). This suggests that raising mice in an EE increases their rearing behavior at this age. We later performed the hippocampus-dependent novel object recognition (NOR) task [41, 42]. We measured the time the mice spent exploring a novel object over a familiar one (Fig. 3E, F). The discrimination indices reveal that at P19-P21 neither the EE mice nor the SH mice demonstrated preference for the novel object (mean DI P19–P21: SH = 0.54 p = 0.38, EE = 0.48, p = 0.72). However, both groups showed preference for the novel object at P25-P27 (mean DI P25–P27: SH = 0.66 p = 0.02, EE = 0.69, p = 0.005). This demonstrates that pre-weaning enrichement does not alter the timing of the acquisition of memory discrimination nor the ability to discriminate between distinct objects.

## Discussion

In light of the strong remodeling of the inhibitory and excitatory network induced by pre-weaning enrichment in the DG [3], we attempted to ascertain whether information processing in the circuit was affected by this plasticity. First, we employed a novel *in-vitro* electrophysiological paradigm to measure the inputs/outputs decorrelation in the DG [31, 32]. By testing different similarity metrics, we first confirmed that in juvenile SH mice the GC performs pattern separation through a high degree of orthogonalization (R and NDP) and low levels of scaling (SF) as shown in previous studies (Fig. 1) [32, 43]. When comparing the similarity metrics recorded from the SH and EE mice, we found that EE slightly but significantly changed two metrics in the opposing direction (SF and the Pearson correlation factor, R) (Fig. 1B). The result suggests that the EE moderately reduces the degree of decorrelation (R) yet increases the levels of scaling (SF) of the inputs in the DG. This finding demonstrates that postnatal experience alters *in-vitro* pattern separation of the GC’s spiketrain. Although the firing pattern in EE mice was unchanged (as observed with firing rate and burstiness, Fig. 1E), changes in SF suggest that the filtering capacity of the DG may be impacted by EE. It is difficult to evaluate the degree to which the changes in DG computation as seen with the spiketrain transformation metrics (R, NDP and SF) relates to behavioral and cognitive changes. It is worth noting that, in a mouse model of temporal lobe epilepsy, the changes in spike train transformation remains relatively small considering the associated mnemonic declines [43]. One possible explanation is that even small changes in R, NDP and SF metrics have a substantial impact on information processing in the circuit. Moreover, while the 10 Hz Poisson train stimulation used in this experiment is physiologically relevant for the DG (Fig. 1) [28], higher frequencies have been reported [44, 45]. An increase in input frequencies leads to enhanced inhibitory conductance [46, 47] and has a strong influence on the processing of the incoming EC signal by GC [32, 47]. Future work is needed to test whether the effect of EE on EC input transformation is more pronounced at specific input frequency. Other putative effects on the transformation of the EC signal may be undetected by our spiketrain protocol. Our previous work observed a ~ 50% increase in excitatory inputs on GC [3] and such increase may enhance coincidence detection [48]. A change in coincidence detection could in turn affect GC’s response to EC inputs, particularly by changing the integration of lateral and medial EC inputs (each stream of inputs encodes distinct information about an animal’s experience [49, 50]). It could also affect subthreshold millisecond coactivity between dentate neurons. Coactivity between GC and interneurons has recently been shown to occur *in-vivo* during successful pattern separation task [51]. It is also possible that the synaptic remodeling in the DG of the EE mice observed in our previous study has little impact in the transformation of the EC inputs at the spiketrain level. Because the encoding of information in the DG depends on the sparsification of the EC activity [16, 52, 53], we assessed whether the pre-weaning enrichment affects the recruitment of the GC during behavior. Our result shows that the exploration of novel objects in the arena increases the number of GC expressing NPAS4 and cFOS. However, the two-way ANOVA analyses show that the EE does not impact observed increases of cFOS + and NPAS4 + . Thus, we can assume the likelihood that a neuron becomes active during the NOE is not affected by the EE pre-conditioning. Other studies have also assessed the effect of an EE on IEG expression in both the amygdala and the hippocampus [54, 55]. Surprisingly, EE led to a reduction of novelty-induced Arc expression in the DG of adult EE rats [55]. The species and age differences could explain the opposite EE effect on IEG expression. Additionally, exploratory behavior during the task may account for the difference between juvenile mice and adult rats. The Arc study was done without assessing the behavior of the animals making it difficult to directly compare their findings with ours. In the present study, the observation that the EE and SH mice have the same exploratory behavior during the NOE task (Fig. 2F, G) supports the conclusion that the recruitment of the GC during the NOE is identical in both SH and EE mice. Taken together, our results suggest that the experience-dependent plasticity taking place in the DG of EE mice [3] has limited effect on the recruitment of the GC downstream of the EC inputs activity (Fig. 2). Based on the present work, we argue that the sparsification of the EC inputs is preserved in mice reared in an EE. More work is needed to further assess whether the transformation of the EC inputs by the DG is affected by EE. The filtering and decorrelation activity of the DG is thought to prevent the overlap of GC ensembles encoding similar, yet different, representations as postulated by the theory of population code of pattern separation [56]. However, sparsification alone cannot explain this phenomenon. Indeed while orthogonality may arise in part by chance due to sparse activity, recent *in-vivo* calcium imaging study calculated that orthogonality is above chance in the DG [57]. It is therefore possible that the synaptic plasticity affecting the circuit in the EE group decreases the overlap of the GC ensembles encoding different contexts or objects while maintaining the same percentage of active cells in the circuit during memory encoding (this would maintain the sparse activity in the circuit while increasing pattern separation). This could take place through a change in novelty induced GC depolarization [58], a change in lateral/feedback inhibition [59, 60] or feedback excitation as well as a change in millisecond timescale co-activity pattern between hippocampal cell [19, 61]. As the temporal co-activity coding emerges as an important mechanism to enable pattern separation [19, 61], it is also important to acknowledge that the time window at which we assess the recruitment of GC using expression of IEG is a few orders of magnitude away from neuronal spiking. Thus, caution must be taken when interpreting results obtained from activity-tagging methods. Future work using *in-vivo* electrophysiology [19, 53] and calcium imaging [52, 62–64] will enable us to decipher the putative role of the experience-dependent plasticity in tuning DG function and possibly enhance pattern separation. Interestingly, EE has been shown in adults in both rats and mice to improve performance in the NOR task [65–68]. Using the NOR and the novel object localization (NOL) tasks, it has also been shown that the ontogeny of these forms of memory discrimination takes place right after weaning [69]. Moreover, in adult, plasticity of the CCK + inputs has been shown to be involved in memory discrimination using fear conditioning protocols [70]. Together, these raise the possibility that the plasticity induced by EE [3] could affect the ontogeny of memory discrimination. However, the present results using the NOR task show that mice reared in an EE do not show a precocious acquisition of memory discrimination. In young animals, both EE and SH mice do not discriminate the novel object (Fig. 3E) while in older juveniles (at P25-27), both groups demonstrate similar discrimination indexes significantly above chance (Fig. 3F). Our result suggests that the synaptic plasticity observed in the hippocampus of animals reared in EE has little effect on the development of the discrimination of dissimilar objects and retention of memory related to these objects. We do not rule out the fact that other forms of memory discrimination are more sensitive to experience and linked to the plasticity induced by EE during the pre-weaning period. To challenge the mice and perform NOR tasks that rely more on pattern separation, the use of very similar yet distinct objects should be done in future studies. Indeed, it was shown that voluntary running from the EE is necessary for memory discrimination in adult mice when two objects are difficult to discriminate [71]. Future work should also use additional memory discrimination tasks taking context into account such as context-dependent fear conditioning, place avoidance and context-dependent NOR. In fact, lesion studies suggest that memory and sensory discrimination involved in the NOR task do not require DG function [72]; however, object-location discrimination tasks as well as other form of memory involving the association of object with context [73–75] are affected by such lesions. Although we strongly believe that DG plasticity must affect the functioning of the circuit, the methods used in this study interestingly show that (i) the decorrelation of the EC inputs by the DG is moderately affected, (ii) the recruitment of GC during exploratory behavior is preserved and (iii) the ontogeny of memory discrimination of distinct object is preserved. These results are in contrast with the massive synaptic remodeling observed in the DG in our previous study. We found about a 50% increase of excitatory synapses in the molecular layer (where EC inputs terminate) and a 50% increase of CCK + synapses in the GC layer [3]. This shows that the circuit has a remarkable ability to remodel the synaptic network while maintaining its computation. Other studies support the idea that dramatic changes in synapse number are not necessarily associated with apparent change in cognitive or sensory performance. For example, decrease in spine density during two consequential monocular deprivation did not directly correlate with eye-specific response [76]. Nonetheless, it remains to be established whether other form of DG computations are affected by EE induced plasticity. Our work focused on a few important computations performed by the DG—circuit-level pattern separation and behavioral similarity discrimination—but these computations only represent a small cross-section of more complex roles that DG might play in memory. Additional work is required to decipher the role of DG synaptic plasticity in juveniles and how it affects circuit function and potentially behavior. Such studies will have tremendous benefit for our understanding of brain disorders and potentially help identify new therapeutic targets. Pre-weaning enrichment has a long-lasting behavioral effect into adulthood [7, 10, 77]. Considering the beneficial effect of early life enrichment against human disorders such as autism and Alzheimer disease [78–80], understanding the effect of an EE on hippocampal circuit function can provide new insight into how environmental factors shape the maturing brain and protects against neurodevelopmental disorders as well as neurodegeneration and cognitive decline in aging adult.

## Material and methods

### Animal husbandry and EE raising

All animal procedures were approved by the University of Nevada Reno Institutional Animal Care and Use Committee, which were in accordance with federal guidelines. Both female and male C57BL/6 mice were used for all experiments. All mice were exposed to a 12 h light/12 h dark cycle with food and water provided ad libitum. The pre-weaning enrichment protocol consisted of placing pregnant female mice at embryonic days 16–19 (E16–19) to enrichment housing 4–7 day before delivery with another female companion. The enrichment environment (EE) cage consists of a large Plexiglas laboratory cage (60 × 45 × 20 cm) containing objects of various shapes, colors, and textures, including plastic houses, tunnels, wood blocks and a running wheel. The different objects and positions were rearranged every other day to maximize novelty. All pups were raised in EE cages from birth to juvenile, while the pups that were referred to standard housing were placed with their female breeder in standard control cages. All experiments were performed on animals between postnatal days 19–21 before weaning.

### Electrophysiology for spiketrain pattern separation

To study pattern separation, we used a method developed by Madar and colleagues to quantify the transformation of the incoming signal in the DG by individual GC (Madar et al., 2019a). SH and PE mice age P19 to P21were anesthetized with isoflurane and decapitated before the cerebral hemispheres were removed and bathed for one minute in ice cold slushy sucrose-based dissection solution containing (in mM): 87 NaCl, 25 NaHCO3, 1.25 Na2HPO4, 2.5 KCl, 7 MgCl2, 10 glucose, 0.5 CaCl2, 1.3 ascorbic acid, 75 sucrose and equilibrated with 95% O2/5% CO2. Horizontal hippocampal slices (300 µm thickness) were obtained using a VF 300-0Z microtome (Precisionary Instruments) and transferred to a recovery chamber with modified artificial cerebrospinal fluid (ACSF) consisting of (in mM): 92 NaCl, 30 NaHCO_3_, 1.2 Na2HPO_4_, 2.5 KCl, 0.5 CaCl_2_, 10 MgSO_4_, 25 glucose, 20 HEPES, 5 ascorbic acid, 2 Thiourea and 3 sodium pyruvic, oxygenized with 95% O_2_/5% CO2. Slices were recovered first for 30 min at 32–34 °C and then for another 30 min at room temperature before recording. Sections were maintained at room temperature for the duration of the experiment (4–6 h). The recordings were performed on putative mature GC located in the outer GC layer. We used a SutterPatch double IPA amplifier (Sutter instrument). Signals were acquired using SutterPatch software (Sutter instrument), filtered at 5 kHZ and sampled at 10 kHz. During the recording, slices were maintained in oxygenized standard ACSF, containing (in mM): 124 NaCl, 24 NaHCO_3_, 1.2 Na2HPO_4_, 2.5 KCl, 2 CaCl_2_, 2 MgSO_4_, 12.5 glucose, 5 HEPES. Whole-cell current-clamp recording of GCs was performed using an internal solution containing (in mM): 140 K-gluconate, 10 EGTA, 10 HEPES, 20 Na-phosphocreatine, 2 Mg-ATP, 0.3 Na-GTP, and 0.1 spermine adjusted to pH 7.3 and 310 mOsm with KOH. The membrane potential of the GCs was monitored in response to stimulation of the perforant path fibers with theta glass pipet connected to a stimulator (A-M System Model 4100). The stimulation protocol consisted of 10 sets of five distinct, 2 s, 10 Hz Poisson trains, delivered every 5 s. These input spike trains were generated using Matlab (R2019a, Mathworks) and followed a Poisson distribution with a similarity between trains set at R_input_ = 0.76 (average Pearson’s correlation coefficient with a bin window of 10 ms). The intensity of the stimulation was determined to obtain a probability of inducing a spike in response to the stimulus in the range of 30%–80%. The level of convergence or separation operated by the circuit was then calculated based on the comparison of the similarity of the inputs spike trains with the similarity of the recorded outputs spike trains. Multiple metrics were used to measure the similarity of the input spike trains and the 50 recorded output traces (10 times 5 inputs). Each metrics used (i.e. R, NDP and SF) informs about distinct features of the spiking pattern such as firing rate, temporal coincidence of the spikes, “orthogonalization” of the signal, and scaling factors; all representing putative neural codes [32]. Results were reported as pairwise comparison of the similarity of the inputs versus those of the outputs (e.g. NDP_inputs_ versus NDP_outputs_). Analysis of covariance (ANCOVA) was used to test statistical significance of the correlation comparison. Because all inputs were generated with a R_input_ = 0.76, the mean of the outputs for each metrics was also compared. In such cases, the statistical analysis was performed using a two-sample t-test. To analyze the firing pattern of the outputs, we calculated the firing rate as defined by the number of spikes per sweep and burstiness, the number of spikes following a single stimulus. We further analyzed the transformation of the input spike trains by the circuit using additional metrics, compactness, and occupancy. These metrics developed by Madar et al. are based on the binning of the spike trains and take into consideration the spikes distribution along the trains (“burstiness” of the trains); compactness taking into account the number of occupied bins in a train whereas occupancy is defined by the number of spikes in the occupied bins. Dispersion metrics can then be calculated by performing a pairwise comparison of the compactness and occupancy for the input and output spike trains of each recording. Generated input trains and data analysis were performed using Matlab (R2019a, Mathworks), and original scripts were generously provided by Dr. Jones’s lab (https://github.com/antoinemadar/PatSepSpikeTrains).

### Behavior

For the novel object exploration (NOE) task, mice were placed in an arena (40 cm*40 cm, Any-box by Stoelting) and allowed to explore and investigate 4 distinct objects placed on the exact same location for every mouse tested. Video recording, collection and analysis was done using ANYMAZE software (Stoelting). For the novel object recognition test (NOR), 5 min habituation was first performed by individually handling each mouse on day 1. The second and third day, mice were placed in the arena and allowed to freely move while being video recorded for 10 min. The data from day 2 were used for the open field analysis (Fig. 3A–D). On the fourth day, a training session was performed, which involved placing two identical objects in the arena, and letting the mouse explore for a total period of 10 min. On day five, one of the familiar objects was replaced by a novel object, and the animal was allowed to explore the arena for 10 min. The arena and objects are thoroughly cleaned and sanitized with EtOH after each use/test and dried prior to the following mouse is introduced. As for the open field and NOE, data were collected and analyzed using ANYMAZE software (Stoelting). Discrimination index was calculated as the ratio of time spent on exploring the novel object divided by the total time exploring both objects.

### Immunohistochemistry and cell counting

90 min after the NOE task, mice were euthanized by transcardial 4% paraformaldehyde perfusion. Brains were removed and postfixed in 1% PFA solution overnight. Leica vibratome was used to section the brain at 90 µµ and sections were then used for immunohistochemistry using free-floating method in 24 well-plates. Sections were first incubated with a blocking solution containing 4% bovine serum albumin, 2% triton and 0.01% sodium-azide for 4 h. Sections were then incubated 24 h at 4 °C with the same blocking solution in which NPAS4 (rabbit monoclonal, Activity Signaling lot #NP41-2) or cFOS (rabbit polyclonal, Synaptic System 226 003) antibodies were diluted at 1/500 and 1/1000 respectively. Sections were then washed in PBS and then incubated overnight at 4 °C with the secondary antibodies (Invitrogen Alexafluor 647) diluted at 1/500 in the blocking solution. Sections are then counterstained with DAPI and mounted with Aqua Poly/Mount (PolyScience). Confocal images were acquired with a Leica SP8 confocal and Imaris software (Oxford Instruments) used to quantify the density of cFOS + and NPAS + cells in the DG. Using Imaris software, the surface tools was applied to the DAPI channel to create a mask defining the GCL area. This mask was then used to identify cFOS + or NPAS + cells using the surface tools. A threshold was applied to all images to solely count brightly labeled cells. Cells with intensity close to the background were not counted as positive. The number of positive cells was divided by the surface of the GCL mask and the mean number of cells per micrometer square was calculated for each group.

### Statistics

All statistical analyses were performed using SPSS and Matlab (Mathworks). Except for pairwise analysis presented in Fig. 1, all data are presented as mean ± SEM. Data were analyzed with parametric tests, including 2-tailed two-sample t-test or two-way ANOVA followed by Tukey post hoc analysis for comparisons of multiple samples. Free-to-use software Jamovi was used for the two-way ANOVA (The jamovi project (2021). *jamovi* (Version 1.6) [Computer Software]. Retrieved from https://www.jamovi.org) For DI (Fig. 3E, 3F), one sample t-test was used to calculate a significant difference from hypothetical 0.5. Sample sizes were not predetermined but are similar to those reported by previous publications in the field [5, 81]. The digital numbers presented within the histogram bars of all figures represent the number of animals per condition (top) and the number of biological replicates (bottom). Individual dots represent the mean for each animal. Statistical details of experiments can be found in the figure legends. p values < 0.05 were considered statistically significant.

## Data Availability

The datasets including immuno-stained images, electrophysiology recordings and video recording used and/or analyzed during the current study are available from the corresponding author on reasonable request.
